# A case report of system configuration issue in medical imaging due to system upgrade– changes in hardware and software

**DOI:** 10.3389/fdgth.2024.1371761

**Published:** 2024-09-02

**Authors:** Md Shafiqur Rahman Jabin, Dianne Wepa, Abdallah Hassoun

**Affiliations:** ^1^ Department of Medicine & Optometry, Linnaeus University, Kalmar, Sweden; ^2^ Faculty of Health Studies, University of Bradford, Bradford, United Kingdom; ^3^ Faculty of Health, Charles Darwin University, Darwin, NT, Australia; ^4^ Region Gävleborg, Healthcare Common Resources, Gävle, Sweden

**Keywords:** picture archiving, patient safety, healthcare quality improvement, software issue, training, system integration, system design

## Abstract

Although the rapid growth in the efficiency of medical imaging is undeniable, the expansion of health information technology (HIT) into medical imaging has not been as seamless or well-integrated as it was thought to be. The socio-technical complexities in medical imaging associated with HIT systems can cause risks to patient harm and inconvenience, both individually and collectively, often in new, unforeseen, and unexpected ways. This study reflects a retrospectively collected single incident report related to medical imaging HIT systems, aiming to develop a set of preventive and corrective strategies. A combination of multiple deductive approaches (existing frameworks), i.e., HIT Classification Systems and 18-step medical imaging process workflow and inductive method (content analysis), were used to analyze the incident. The incident was identified as a “system configuration”-related software issue, contributed by system upgrade– changes in hardware and software. The incident was determined to occur during steps 10–12, i.e., “study selection and retrieval,” “calling up of patient's referral,” and “image review and interpretation,” causing severe disruptions in the clinical workflow for several weeks. We propose 16 preventive and corrective strategies grouped under four key areas based on the socio-technical aspects associated with HIT systems. The key areas are (i) preparation and integration for upgraded systems, (ii) training for medical imaging specialists, (iii) contingency planning/immediate backup system, and (iv) system design and configuration. These strategies are expected to help healthcare staff, analysts, reporters, researchers, and relevant stakeholders improve care delivery and patient safety in medical imaging in the context of any system upgrades.

## 1 Introduction

Over the last four decades, medical imaging modalities such as x-rays, CT scans, MRI, and techniques like Deep Learning (1, 2) and Federated Learning (2) have progressed rapidly alongside the immense advancement of modern medicine (1–3). Undeniably, the rapid growth in the efficiency of medical imaging, largely driven by recent advances in Deep Learning and Federated Learning and the use of larger and more diverse training sets, is a testament to collective efforts and achievements in modern medicine (4). It is crucial that the efficiency of medical imaging meets the expectations of the healthcare staff, whose additional hours spent with dysfunctional devices and systems not only lead to frustration but also underscore the urgent need for improvement (4, 5).

Meanwhile, healthcare quality improvement and patient safety have increasingly been the top priority over the last 40 years, with the progressive realization that the delivery of care in medical imaging can itself harm patients (6). It is also important to remember that the person who is to be imaged is often in a vulnerable state and out of their comfort zone. The role of medical imaging technicians is not just to produce a high-quality image but also to facilitate patient care throughout the imaging process (7). Medical imaging in the healthcare system now comprises truly sociotechnical complexities despite its many benefits and strengths. It can also cause risks to patient harm and inconvenience, both individually and collectively, often in new, unforeseen, and unexpected ways (6).

Health information technology (HIT) has been defined as: “hardware or software that is used to electronically create, maintain, analyze, store, receive (information), or otherwise aid in the diagnosis, cure, mitigation, treatment or prevention of disease and that it is not an integral part of an implantable device or medical equipment” (1, 2, 8). The merging of medical imaging and HIT systems, such as Radiology Information Systems (RIS) and Picture Archiving and Communication Systems (PACS), has been introduced to modern healthcare to improve efficiency (9). The HIT systems have been promoted to streamline operations and optimize available technology to be safe and effective (4). Nevertheless, the expansion of HIT into medical imaging has not been as seamless or well-integrated as it was thought to be.

Multiple issues are associated with HIT systems in socio-technical contexts, such as hardware and software-related issues, system upgrades—hardware and software modifications (6, 9). Software-related challenges associated with HIT systems can be of different types, such as software functionality, system configuration (problems with default settings), increased volume of transactions, interface with software systems or components, and viruses/malicious attacks (10–12). The hardware issues may involve “device down or slow”, issues related to data capture or output peripheral, data storage and backup, and power failure (6). In addition, a study on the analysis of 436 medical imaging-related HIT incidents indicated that around 10% of the total sample was associated with “system upgrades”. These upgrades involved upgrading the PACS software, scanner software, server software, and RIS system. The consequences of these issues could range from patient inconvenience to patient harm or workflow interruptions to single or multiple facilities or even the entire healthcare (5, 6, 13, 14).

For example, Jabin et al. demonstrated an analysis in 2019 of how 436 HIT incidents occur infrequently at each of the 18 steps of the imaging workflow process, contributed by either human or technical factors, with the consequences of outcomes sporadically reported—often not evident or not yet occurred at the time of (14). Several outcomes were associated with the imaging workflow, such as interruptions in patient treatments, patient inconvenience, delays in delivery of care, and risks to patient safety, including repeated or unnecessary radiation to patients. The patient outcomes were misdiagnosis, missed diagnosis, and delayed diagnosis, whereas the staff and organisational outcomes were delayed reporting and confusion among imaging staff, particularly during image review and interpretation (5, 6, 13, 14). The major problem of these incidents affecting the workflow is that once a wrong piece of information or document is initiated into any HIT system, an “automation bias” tends to consider it correct (15, 16).

Collecting information after something goes wrong, such as incident reports, may help understand the underlying mechanism for how and why they go wrong. This necessitates qualitative research—collecting qualitative data in the form of free-text narratives or anecdotes. This form of study, in turn, allows us to understand the healthcare context, identify and characterize the risks, contributing factors, consequences of the risks posed, and actions taken to manage the risks (9). The approaches to analyzing the qualitative free-text narratives are inductive (extracting themes in the narratives) (17) and deductive techniques. The deductive method comprises the classification of the critical aspects of the qualitative data by feeding them into an existing framework, such as the HIT Classification System (HIT-CS) (18) and 18-steps of the medical imaging process workflow (14). The HIT-CS was developed to map a conceptual framework for understanding and classifying things that go wrong in healthcare associated with HIT systems (19). In comparison, the 18-step imaging process workflow was framed to inform the analysts where preventive and corrective strategies should be addressed.

Since limited research has been conducted on system configuration issues in medical imaging, there is an urgent need for qualitative exploration of such problems. Therefore, it is essential to analyze retrospectively collected medical imaging incident reports to illuminate patient safety issues in Swedish healthcare and characterize the problems associated with their human and system-based causal factors. This report will address some practically applicable insights for medical imaging professionals, researchers, and analysts to understand where preventive and corrective strategies could be addressed to better support the issues associated with system upgrades– changes in hardware and software. The report explores the following research questions:

1. What is the reported issue involving the HIT system used in medical imaging?
2. What were the contributing factors and consequences of that HIT issue related to medical imaging?
3. What potential preventive and corrective strategies would be used to reduce the risks associated with the HIT systems used in medical imaging?

## 2 Methods

This is a qualitative study in which data was collected from an organization responsible for the healthcare incident repository. The data, i.e., free-text narratives, was then analyzed using both multiple deductive and inductive approaches.

### 2.1 Data collection

As presented in Box 1, this case report is a medical imaging-related HIT incident report extracted from the reidarMTP, i.e., an electronic database for registering incidents related to any medical devices and/or their use in the healthcare environment. The incident has been presented in three fields: “incident description,” i.e., reported by an anonymous healthcare professional, “summary of cause investigation,” i.e., an internal investigation analysis after reporting the incident, and summary of measures, i.e., actions taken to manage risks. The reports are anonymous and freely available for quality improvement, education, and training to all healthcare professionals. The database is operated by a voluntary association of clinical engineering departments in Swedish hospitals and is managed by certified trained staff (20, 21).

The reports are generally categorized into multiple different fields, comprising various sets of information. The first category includes the date, day, and time of events, as well as an incident description with a short subject line, such as “problems in the imaging workstation”. The second category is about the type of products involved in the incident, such as product name, manufacturer, software version, serial/batch number, etc. The third category entails a thorough investigation, including a summary of cause investigation, a summary of actions, and a summary of follow-up. The final category involves classification or risk assessment, including risk of medical damage and underlying cause (22). The incident in Box 1 has been filtered and illustrated in three fields: “incident description,” which was reported by anonymous healthcare staff, “summary of cause investigation,” i.e., an internal investigated narrative of the reported incident, and a “summary of measures”. The name of the software program/product has been kept anonymous, and the incident has been filtered before being presented in Box 1.

The report was provided in Swedish and later translated into English by a linguistic expert who has proficiencies in both languages, i.e., Swedish and English. To ensure the accuracy and credibility of the report, the technical nature of the content was carefully considered with the help of consensus throughout the translation process by the linguistic expert and the principal investigator.

**BOX 1 This medical imaging-related HIT incident was reported to the reidarMTP by an anonymous user showing responses to the following categories of information.**

**Incident Description**

Digital imaging systems, i.e., PACS, had been struggling for several weeks. We could not see or display examinations; the work was slow, and we had to improvise different solutions and try to find examinations in image viewers that we were not used to (and so on). It was not acceptable from a work environment point of view, and there was a risk that patient safety had been threatened. Today, there were two emergency patients who did not have their images available before acute surgery.

**Summary of Cause Investigation**

In this case, general problems occurred with the PACS workstation, which was previously used within the hospital. In connection with a change of hardware, server operating system, and database manager, large deteriorations in the systems’ performance occurred.

After several weeks of troubleshooting, the supplier found a configuration that was missing from the application. This missing configuration caused the system to spend a lot of time updating image files without really needing to. This, in turn, resulted in examinations being locked and inaccessible. After the setting was adjusted, the systems worked better.

The reason was also connected to the fact that a planned failover (change of active data center) in the image archive was carried out at 11:55 on the same day. Soon after, error reports began to come in, where it appeared that a number of newly produced examinations were not available.

After various troubleshooting by the supplier during the afternoon, it turned out that there were a number of examinations that had not been replicated (copied) from the previously active part of the image archive to the part that was made active at 11:55. Synchronisation work began and continued approximately until midnight. Information is posted on the intranet.

Part of the reason the vendor was unaware that the two halves of the image archive were not fully synchronized since one of the tools showed 100% synchronized data, but it was actually a rounded value.

**Summary of measures**

Adjustment of configuration in X and synchronization of the imaging archive.

### 2.2 Data analysis

The following incident was analyzed using both inductive (content analysis) and deductive approaches (existing framework). The deductive approach included multiple existing frameworks—an existing classification system proposed by Magrabi et al., i.e., the HIT-CS (18), and a framework used by Jabin et al., i.e., the 18-step of medical imaging process workflow (14). The HIT-CS is helpful in deconstructing HIT-related incidents and categorizing types of problems, contributing factors, and consequences to extract meaningful information (18). The 18 steps of the medical imaging workflow-based classification system are useful as they orientate the reporters, researchers, and analysts to the tasks at each stage. It also helps inform the analysts as to where preventive and corrective strategies could be addressed to overcome the specific problem in question (14).

The HIT-CS was used to identify the type of system issue and the type of consequences associated with the incident. The 18-step process workflow, combined with the content analysis, was used to understand the underlying mechanism of what went wrong, why it went wrong, and at which stage of the imaging process it went wrong. The ultimate purpose of using all these analyses was to devise a set of preventive and corrective strategies that could eventually mitigate the risk of similar incidents occurring in the future.

## 3 Results

Using the HIT-CS, the incident was categorized as a software issue (technical problem), i.e., “system configuration”, and the consequence of the incident was classified as an “incident with noticeable consequence but no patient harm”.

**Figure 1:**
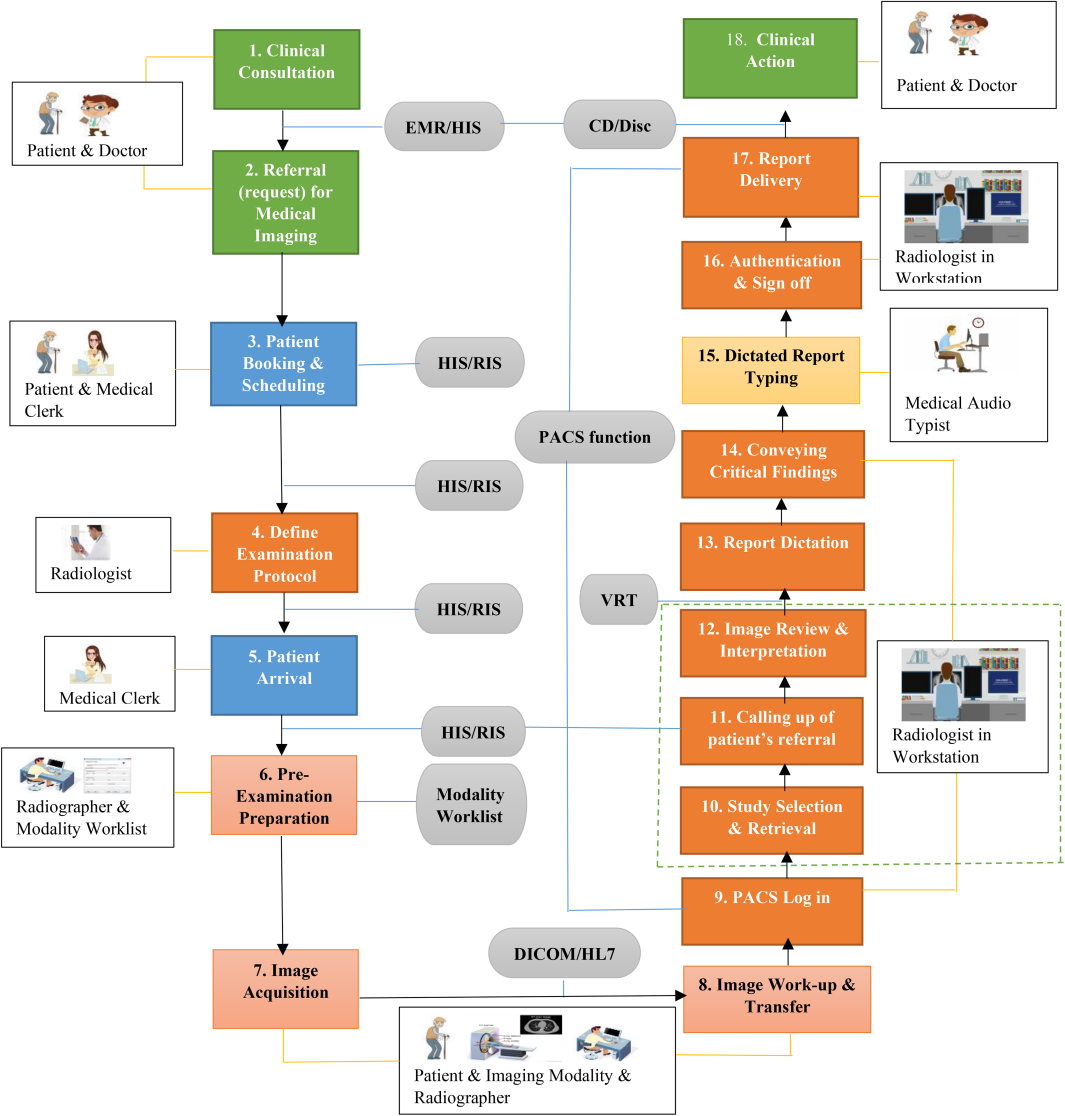
Medical imaging process workflow showing the 18 sequential steps and the HIT systems by which they are mediated.

Using the framework of 18 steps medical imaging process workflow, the incident was identified to occur during steps 10–12 (as indicated by the dotted lines in Figure 1), i.e., “study selection and retrieval”, “calling up of patient's referral”, and “image review and interpretation”.

Using the content analysis, the contributing factors for the incident were changes in hardware and software, such as the database manager and server operating system. The mitigating factor was multiple improvised solutions to retrieve the examinations from the image viewers; however, those improvised solutions were not clearly indicated in the incident description. Although the incident did not cause any harm to patients directly, two emergency patients were found to be at risk of patient safety since their images could not be retrieved in a timely manner. Therefore, the patient outcome was delay in patient treatment, and the organizational outcome was determined to be severe disruptions in the clinical workflow for several weeks. The actions taken to manage the risk were a correction of the missing configuration and synchronization of the PACS.

## 4 Discussion

Although medical imaging HIT systems have improved effectiveness and efficiency, their design, use, and implementation can negatively impact patient care and safety (6, 14). Any changes in or errors due to HIT systems can even contribute to regular workflow interruptions (14). Several pieces of evidence suggest that software-related challenges are common phenomena in various types of HIT systems used in modern medicine, such as e-prescribing systems (10), patient information systems (11), and medical imaging systems (6, 13). These issues can trigger serious consequences, ranging from staff organizational outcome (5) to patient inconvenience (13) and patient harm (5, 23). Among all software issues, software functionality, and system configuration have been the most common software-related challenges (10, 11), including in medical imaging HIT systems (6).

Jabin et al., in 2023, reported different types of system configuration issues, such as “system not designed to support the decision” and “system not designed to give any warning/alert” (11). These issues posed severe risks to patient safety, which were later escalated to maximum severity and priority to meet the required system criteria. On the other hand, multiple studies reported that even a perfect system configuration might become challenging due to changes in system environments or workloads, such as hardware changes, environment changes, resource exhaustion, and software upgrades (24, 25). Hardware changes comprised 18% of the root cause of configuration errors (25).

In this report, we examined things that had gone wrong with respect to medical imaging as a basis for devising a set of preventive and corrective strategies for managing similar issues in the future. It is of utmost importance to overcome the ongoing socio-technical challenges that healthcare face in their daily routine, particularly in the context of medical imaging HIT systems. Therefore, we propose a set of strategies through the lens of socio-technical aspects associated with HIT systems, the reflections arising from the literature and the findings, and stakeholder engagement (led by authors) comprising specialists in medical imaging from the Region Gävleborg, The proposal includes 16 preventive and corrective strategies, which are grouped under four key areas. The key areas are (i) preparation and integration for upgraded systems, (ii) training for medical imaging professionals, (iii) contingency planning/immediate backup systems, and (iv) system design and configuration. We believe these recommendations will help healthcare staff, analysts, reporters, researchers, and relevant stakeholders to improve the delivery of care and patient safety in medical imaging in the context of any system updates or changes. The details of these strategies are presented in Box 2.

It is important to note that this study has a few limitations that may impact the interpretation of the findings. For instance, the study does not follow a protocol for the usual qualitative method used in medical imaging research, such as observation or interviews. Understanding these limitations is crucial for a comprehensive assessment of the research (26–28). The major limitation is that it is a retrospectively collected single anonymous incident report; therefore, it was not possible to follow up with the reporter to extract more details about the event. Moreover, data collection from voluntary incident databases has inherent limitations with the accuracy and specificity of reported data, combined with limited content knowledge, i.e., reporters’ lack of expertise in HIT systems and technologies. However, this limitation was overcome by utilizing the additional fields of information, “summary of cause investigation” and “summary of measures.” Moreover, the incident was scrutinized through multiple lenses—two deductive approaches and an inductive method. This helped obtain a detailed picture of what went wrong and how it went wrong and devise a set of preventive and corrective strategies to overcome such future configuration issues in medical imaging.

The conclusion based on the results of this single incident should be treated with caution, as this case report does not offer any insights into quality improvement interventions or how to measure their effects. One must remember that the risks to patient safety existed even after developing and implementing various radiological interventions, such as the Correct Patient, Correct Site, and Correct Procedure (3Cs) Protocol in 2004 (29). This is mainly because the volume and complexity of the workload in radiology practice have also increased; for example, the daily average volume of medical imaging examinations read by radiologists has increased sevenfold in the last 7 years (29). Therefore, the complications in workload management in this complex sociotechnical system add a layer of other obstacles (3). Moreover, thousands of patients are processed, transported, treated, and examined by hundreds of radiologists and radiographers in daily clinical practice, and the risks for such failures are enormous. Notwithstanding these limitations, the findings and devised preventive and corrective strategies can be generalized and considered as alerts to inform healthcare digitalization and pertinent elsewhere for patient safety and quality improvement studies. Our study's findings, which establish a clear connection between HIT system issues and clinical outcomes, are significant. They not only contextualize the study's significance but also provide a crucial direction for future research efforts. The ultimate purpose of using all these analyses was not just to understand what went wrong and how it went wrong but also to proactively devise a set of preventive and corrective strategies that could eventually mitigate the risk of similar incidents occurring in the future.

**BOX 2 Preventive and corrective strategies to mitigate and manage the risk of medical imaging-related HIT incidents.**

**Preparation and integration for upgraded systems**

▪ Establish close cooperation between the vendors of both the new and “legacy” systems to understand the needs of the facility or service through extensive ongoing consultation and advice-seeking and obtain the right systems in the first place
▪ Work closely in association with vendors to ensure proper and applicable integration of multiple medical imaging systems, such as RIS and PACS, in the facility
▪ Perform robust testing or regression protocol in the system development phase prior to system deployment to avoid or mitigate the occurrence of software functionality and system configuration issues
▪ Plan carefully for any system (hardware and software) changes, local fixes, and system transition to mitigate disruption to regular workflow and ensure contingency plans (see below)

**Training for medical imaging professionals (radiologists and radiographers)**

▪ Train imaging professionals with adequate paid time, jointly organized by healthcare organizations and HIT vendors prior to implementation or deploying any new system
▪ Provide radiologists and radiographers training updates as a refresher and following any hardware changes, environment changes, resource exhaustion, and/or software upgrades

**Contingency planning/immediate backup system**

▪ Ensure immediate backup system and contingency procedures are part of any contract for any high-stakes operations, such as medical imaging in healthcare
▪ Establish sufficient escalation procedures to deal with any new and unforeseen issues that may potentially cause patient harm
▪ Arrange timely access to appointed IT personnel who are adequately trained in all facets of HIT use, monitoring, evaluation, and optimization
▪ Set up a robust mechanism for communicating any unexpected downtimes to all healthcare professionals in the facility

**System design and configuration**

▪ Design and configure medical imaging HIT systems, such as RIS and PACS, so that they are interoperable and coordinate with each other
▪ Design standard user-interface features and functions and develop well-established standards for safety-critical software
▪ Develop and design the HIT systems that fit with the clinical workflow
▪ Synchronise terminologies, exposure indicators, proprietary coding systems, and information systems while implementing or operating systems from various vendors
▪ Ensure decisive information is displayed for decision-support interventions using standardized terminologies and color schemes
▪ Secure access to previous images—prefetching algorithms and display protocols (as mentioned above)

## 5 Conclusion

Although medical imaging efficiency has improved, if those HIT systems are not supported by adequate contingency planning or backup system, appropriate system integration, design, and configuration, unforeseen consequences such as delays, corruption of information, workflow disruption, and patient harm can ensue. Therefore, collecting information after they have gone wrong should be a routine part of clinical practice to provide a basis for improvements for preventing issues and improving such practice. However, an ongoing discussion should be carried out about general HIT problems related to system upgrades– changes in hardware and software.

## Data Availability

The original contributions presented in the study are included in the article, further inquiries can be directed to the corresponding author.
